# Supplementary material for: Socioeconomic Inequalities in Women’s Undernutrition: Evidence from Nationally Representative Cross-Sectional Bangladesh Demographic and Health Survey 2017–2018
Source: Int J Environ Res Public Health. 2022 Apr 13;19(8):4698. doi: 10.3390/ijerph19084698 (PMC9031436; doi:10.3390/ijerph19084698)
Supplement: Supplementary file 1 [file ijerph-19-04698-s001.zip › ijerph-1664532-supplementary.pdf]

Figure S1. Division-wise prevalence of undernutrition in urban and rural settings

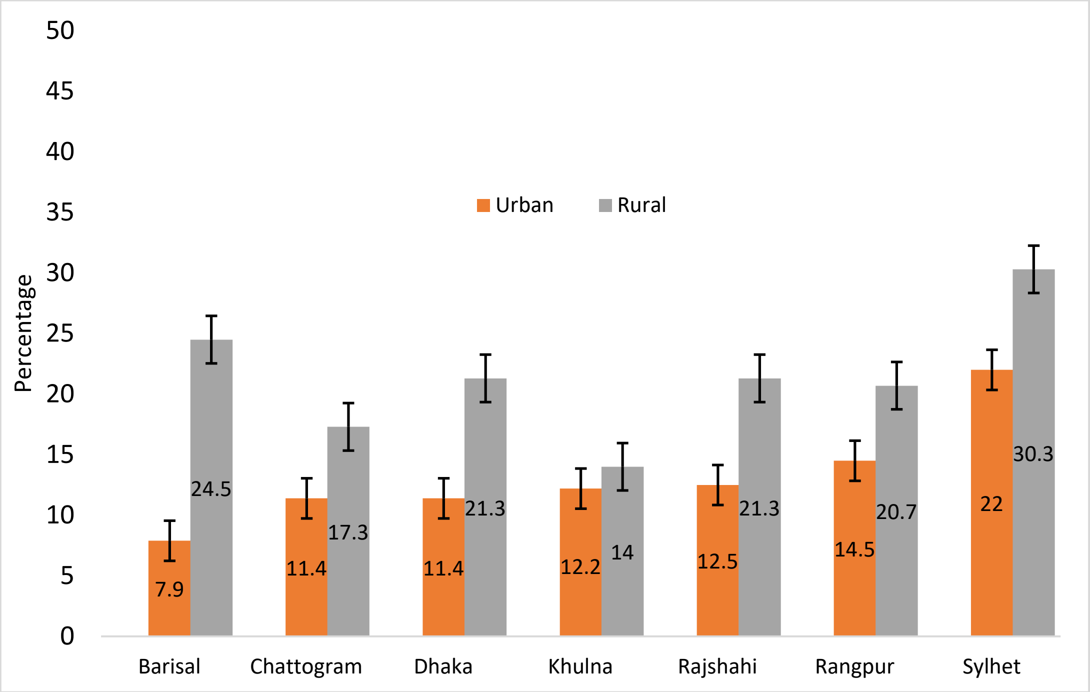

Table S1. Prevalence and association of undernutrition by sample characteristics

Characteristics	n	%	95 % CI	p-value
**Number of a household member**				
<5	990	11.2	10.3, 12.0	0.191
≥5	1377	11.9	11.1, 12.6	
**Age of respondent**				
15-19 years	432	21.9	19.9, 24.1	<0.001
20-24 years	504	14.0	12.8, 15.2	
25-29 years	365	9.5	8.4, 10.6	
30-34 years	297	8.6	7.5, 9.8	
35-39 years	262	8.4	7.3, 9.6	
40-44 years	247	10.3	8.9, 11.9	
45-49 years	260	11.3	9.9, 12.9	
**Respondent's education**				
Illiterate	3160	15.0	13.6, 16.5	<0.001
Primary	855	12.8	11.9, 13.8	
Secondary or higher	997	9.7	9.0, 10.4	
**Respondent's marital status**				
Others (widow/divorced/separated)	194	15.0	13.0, 17.3	<0.001
Married	2173	11.3	10.7, 12.0	
**Respondent's current employment status**				

Unemployed	1142	10.5	9.8, 11.2	<0.001
Employed	1225	12.7	11.9, 13.6	
**Number of living children**				
No child	347	16.2	14.5, 18.0	<0.001
One child	631	13.5	12.4, 14.7	
Two children	587	9.6	8.8, 10.5	
Three or more children	802	10.6	9.8, 11.5	
**Media exposure**				
No	1150	16.0	15.0, 17.1	<0.001
Yes	1217	9.2	8.7, 9.8	
**Type of place of residence**				
Urban	642	8.5	7.7, 9.4	<0.001
Rural	1725	12.7	12.0, 13.5	
**Wealth quintile**				
Poorest	766	19.8	18.3, 21.3	<0.001
Poorer	626	15.5	14.0, 16.5	
Middle	423	10.2	9.2, 11.4	
Richer	359	8.8	7.8, 9.8	
Richest	193	4.3	3.6, 5.1	
**Administrative division**				
Barisal	225	11.1	9.5, 13.1	<0.001
Chattogram	208	7.5	6.3, 8.9	
Dhaka	259	9.4	8.2, 10.7	
Khulna	262	10.6	9.2, 12.1	
Mymensingh	364	17.5	15.4, 19.8	
Rajshahi	295	12.4	10.9, 14.0	
Rangpur	324	13.9	12.0, 16.1	
Sylhet	430	20.8	13.4, 23.6	
